# Interleukin-19 Aggravates Pulmonary Fibrosis via Activating Fibroblast through TGF-*β*/Smad Pathway

**DOI:** 10.1155/2022/6755407

**Published:** 2022-03-03

**Authors:** Yu Wang, Sibo Sun, Kai Wang, Mingjiong Zhang, Min Li, Yumin Zan, Qiqing Huang, Shuangshuang Wu, Weihong Zhao, Wei Xu, Jianqing Wu

**Affiliations:** Jiangsu Provincial Key Laboratory of Geriatrics, Department of Geriatrics, The First Affiliated Hospital of Nanjing Medical University, Nanjing, China

## Abstract

**Background:**

Idiopathic pulmonary fibrosis (IPF) is a progressive and fatal interstitial pneumonia disease with no cure. Communication between injured cells is triggered and maintained by a complicated network of cytokines and their receptors. IL-19 is supported by increasing evidences for a deleterious role in respiratory diseases. However, its potential role in lung fibrosis has never been explored.

**Methods:**

Bioinformatic, immunohistochemistry and western blot analysis were used to assess the expression of IL-19 in human and mouse fibrosis lung tissues. CCK-8, transwell and flow cytometry assay were utilized to analyze the effect of IL-19 on biological behaviors of lung fibroblasts. Histopathology was used to elucidate profibrotic effect of IL-19 in vivo.

**Results:**

IL-19 was upregulated in fibrosis lung tissues. IL-19 promoted lung fibroblasts proliferation and invasion, inhibited cell apoptosis, and induced differentiation of fibroblasts to the myofibroblast phenotype, which could be revised by LY2109761, a TGF-*β*/Smad signaling pathway inhibitor. Furthermore, we found that IL-19 aggravated lung fibrosis in murine bleomycin-induced lung fibrosis.

**Conclusions:**

Our results imply the profibrotic role for IL-19 through direct effects on lung fibroblasts and the potential of targeting IL-19 for therapeutic intervention in pulmonary fibrosis.

## 1. Introduction

Idiopathic pulmonary fibrosis (IPF) is an age-related and progressive disease with no cure. Incidence of IPF has risen over time. It is reported to 2.8–18/100000 people per year in Europe and North America while 0.5–4.2/100000 in Asia and South America [1]. Mortality of IPF is very high, with a median survival time of 2–4 years from diagnosis [2]. IPF is a predominant type of interstitial lung disease (ILD), characterized by chronic inflammation and interstitial fibrosis. Genetic susceptibility, environmental risk factors and exposures can cause repetitive local microinjuries to the lung tissue and the vascular system, which can trigger a cascade of inflammatory responses and fibrosis [3]. Chronic inflammation is considered as a common hallmark of fibrosis diseases [4]. Injured intrinsic and immune cells contribute to the sustainment of chronic inflammation and augment extracellular matrix (ECM) generation through releasing widespread inflammatory cytokines and growth factors [5].

IL-19, a member of the IL-10 cytokine family, is generated by immune cells, epithelial cells, and vascular structural cells [6]. The function of IL-19 is confusing and often contradictory depending on the organization and disease [7]. IL-19 plays multiple roles in several human diseases and their animal models, such as cardiovascular disease [8], inflammatory bowel disease [9], psoriasis [10], rheumatoid arthritis [11], acute kidney injury [12], and breast cancer [13]. In respiratory diseases, IL-19, which is reported involved in inflammatory responses and causing pulmonary injury by activating lung epithelial cells [14], is positively associated with the progression of asthma [15] and chronic obstructive pulmonary disease (COPD) [16]. The immunoregulatory cytokine IL-19 holds promise as new treatment and prevention [17]. Targeting the IL-19 signaling might be a new target for therapeutic intervention in chronic asthma [15]. However, the impact of IL-19 on the development of IPF has never been explored.

Here, we investigated IL-19 expression in human and mouse lung fibrosis tissues and the effect of IL-19 on lung fibroblasts as well as the possible mechanism. Then, we focused on role of IL-19 on wild-type mice and bleomycin(BLM)-induced pulmonary fibrosis mouse models. Overall, our study highlights the role of IL-19 on pulmonary fibrosis in vitro and vivo and proposes a new insight for future research and provides a promising management strategy for treating pulmonary fibrosis.

## 2. Materials and Methods

### 2.1. Bioinformatics Analysis

Gene expression profiles for two datasets (GSE77326, GSE2051) were obtained from the Gene Expression Omnibus (GEO) database (http://www.ncbi.nlm.nih.gov/geo/). The mRNA samples were obtained from GSE2051, comprising of 11 lung tissues of patients with IPF and 13 normal lung tissues samples, and GSE77326, comprising of 6 bleomycin instilled mouse lung tissues and 6 Sham group mouse lung tissues. Samples were subjected to gene expression profiling to determine the different expression profiles between IPF and normal lung tissues. The data sets were processed using the GeoR2 software.

To identify significantly dysregulated biological pathways of IL-19 in IPF, the GSEA was performed by GSEA 4.0 (http://www.gsea-msigdb.org/gsea/index.jsp) under functional annotations of the Kyoto Encyclopedia of Genes and Genomes (KEGG) database (https://www.genome.jp/kegg/). All the genes in each dataset were submitted to cluster profiler, with the permutation number and the minimum gene set size set as 10000 and 10, respectively. The significance level was set as *FDR* < 0.05.

### 2.2. Primary Mouse Lung Fibroblasts Isolation and Cell Culture

Primary mouse lung fibroblasts were prepared as follows [18]: (1) lung tissues obtained from 6–8 weeks old C57BL/6 mice were perfused with 10–20 ml PBS into the right ventricle until the lungs were blood flushed and had a white appearance. (2) The tissue was cut into very small pieces using surgical scissors and then incubated with 0.5 ml of collagenase I (final concentration, 1000 U/ml) (Biofroxx) at 37°C for 30 min. (3) Centrifuge at 1000 g for 5 min, discard the supernatant. (4) Then add 500 *μ*l 0.25% trypsin-EDTA to digest tissues at 37°C for 10 min. (5) After centrifuge, plate the suspension into a 100 mm cell culture dish and adherent cells for 1 hour at 37°C, then discard the supernatant.

The human embryo lung fibroblast cell (HELF) was purchased from iCell Bioscience (Shanghai, China). All cells were cultured in DMEM (11995-065, Gibco) supplemented with 10% fetal bovine serum (FBS, 10099141, Gibco) and 1% penicillin-streptomycin (SV30010, Hyclone) in an atmosphere at 37°C and 5% CO_2_. Human and mouse IL-19 and TGF-*β*1 were purchased from GenScript (Nanjing, China). LY2109761 was obtained from Selleck Chemicals (Houston, USA), solubilized in dimethyl sulfoxide (DMSO).

### 2.3. CCK-8 Assay

The abilities of cell proliferation were assessed by CCK-8 assay (HY-K0301, MCE). HELF and primary mouse lung fibroblasts were placed into plates and cultivated in incubator for 0 h, 24 h, 48 h, and 72 h. Then cells were interacted with CCK-8 solution at 37°C for 2–4 h, and the absorbance at 450 nm was measured.

### 2.4. Transwell Assay

The abilities of cell invasion were evaluated by transwell assay. Cells were added to the upper chambers (Corning, USA) and incubated with stimulation for 72 h while the lower chambers were incubated with DMEM medium containing 10% FBS. Fixed cells migrating into the lower chamber with methanol and stained with crystal violet (C0121, Beyotime). After washed by PBS, cells migrating through the membrane were stained and counted by microscopy (Nikon).

### 2.5. Apoptosis Assay

Apoptosis was determined by Annexin V-FITC staining and analyzed by flow cytometry. The apoptosis of fibroblasts were evaluated at baseline and after treatment with H_2_O_2_ and IL-19. The Annexin V-FITC/PI apoptosis detection kit (A211-02, Vazyme) was used to evaluate the ratio of apoptotic fibroblasts, and the apoptosis was assessed by FACS Calibur Flow cytometer.

### 2.6. Western Blot Assay

Tissues and cells were lysed with RIPA buffer containing protease inhibitors. Load and separate equal amounts of proteins on SDS-PAGE gels. Transfer the proteins to a PVDF membrane after electrophoresis, block membrane in 5% skimmed milk and incubate overnight with the primary antibody (IL-19 (ab154187, Abcam), *α*-SMA (ab7817, Abcam), Collagen I (ab260043, Abcam), TGF-*β*1 (ab215715, Abcam), Smad2/3 (ab202445, Abcam), pSmad3 (ab52903, Abcam), and GAPDH (60004-1-Ig, Proteintech)). Wash the membrane three times with 0.1% tween phosphate buffer solution (PBST) for 10 minutes each time and then incubate the membrane with goat anti-mouse or anti-rabbit for one hour at room temperature. Protein bands were analyzed by ImageJ software. The relative grey values of the target protein to the GAPDH bands were calculated to determine the change in protein expression.

### 2.7. Immunofluorescence

Cells were fixed with 4% paraformaldehyde for 15 minutes, permeabilized with Triton X-100 (1139ML100, Biofroxx) for 20 minutes, blocked with 1% BSA (4240GR100, Biofroxx) for 30 minutes, and incubated overnight with specific primary antibody. Thereafter, probe cells with conjugated goat anti-mouse IgG (*H* + *L*) (SA00013-1, Proteintech) or rabbit IgG (*H* + *L*) (SA00013-2, Proteintech) at room temperature in the dark for one hour. Counterstain cell nuclei with DAPI (C1002, Beyotime). Then observe cells under a fluorescent microscope.

### 2.8. Induction of Pulmonary Fibrosis In Vivo

Male C57BL/6 mice weighing 18–22 g were divided into 4 groups: (a) saline group (*n* = 6); (b) BLM (*n* = 8) group; (c) IL-19 (*n* = 8) group; (d) *BLM* + *IL* − 19 group (*n* = 10). On day 0, mice were anaesthetized by Avertin (125 mg/kg, i.p.). After sterilizing the neck using betadine, make a 1 cm midline incision with sterile scissors and insert the microinjector into the exposed trachea, inject the saline/bleomycin (2.5 mg/kg, HY-17565A, MCE)/IL-19 (200 ng/kg, Z03113, GenScript) during a single inspiration. After withdrawing the needle, close the incision with suture clips. Place the animal on warmer pads to allow recovery [19]. The peripheral bloods were collected on day 21 to determine the levels of IL-19 by Mouse IL-19 ELISA KIT (SEKM-0020, Solarbio). The lung tissues were collected for histopathological examination, hydroxyproline analysis [20] and western blot analysis.

### 2.9. Histopathology and Immunohistochemistry

The lung lobes were fixed in 4% buffered paraformaldehyde and immobilized in paraffin for H&E or Masson trichrome staining. The severity of fibrosis in lung tissues was semi-quantitatively assessed by Ashcroft scoring system [21]. Histological changes are assessed on the basis of alveolar wall thickening, inflammatory disorder, and the extent of collagen deposition. Immunohistochemistry was performed by incubating tissue sections with primary antibodies (IL-19 and *α*-SMA) as described in previous protocol [22].

### 2.10. Statistical Analysis

Data are reported as the *mean* ± *SEM*. Difference between two groups comparing experimental groups was analyzed using Student's *t*-test, while more than two groups comparison using one-way analysis of variance (ANOVA). Analysis of data was conducted by GraphPad Prism 8 software. *P* < 0.05 was considered as statistically significant difference.

## 3. Results

### 3.1. IL-19 Is Upregulated in Human and Mouse Lung Fibrosis Tissues

**Figure 1:**
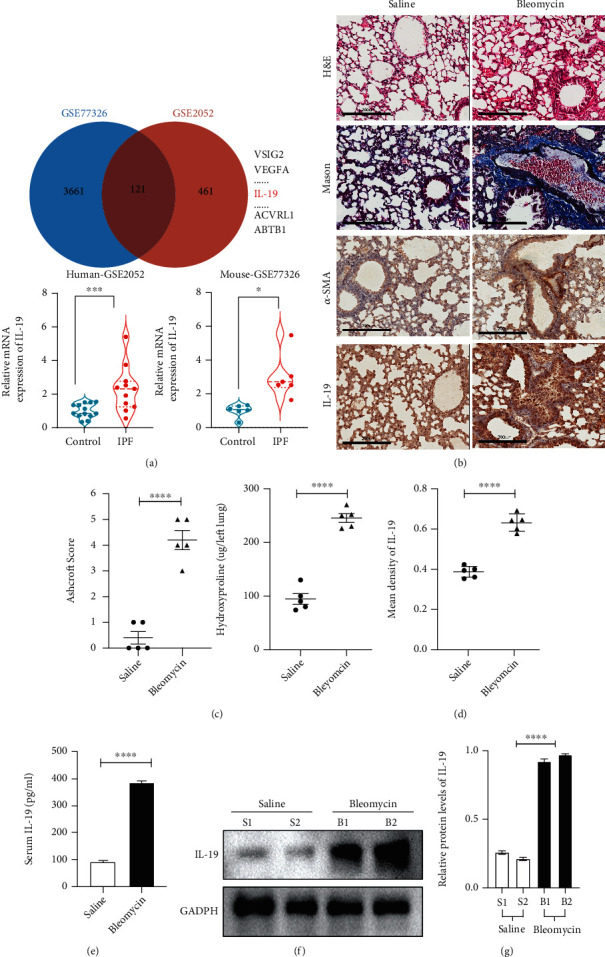
Expression of IL-19 is upregulated in human and mouse lung fibrosis tissues. (a) The gene expression profiles of two independent datasets (GSE77326, GSE2051) were used to assess the differential gene expression in lung fibrosis tissues. 121 gene expressions were higher both in human and mouse lung fibrosis tissues than controls, and mRNA expression of IL-19 in IPF was significantly higher than that in control. (b) Representative images (taken at ×20 magnification) of H&E, Masson's trichrome, and immunohistochemical staining of IL-19 and *α*-SMA in BLM-induced murine lung fibrosis tissues and controls. Scale bars, 200 *μ*m. (c) Quantification of the degree of pulmonary fibrosis according to the modified Ashcroft scale and pulmonary hydroxyproline content. (d) Amount of IL-19 was quantified using automated image analysis of the IL-19 staining. (e) Serum IL-19 expression was analyzed by ELISA in the BLM-induced mice and controls. (f) IL-19 protein expression in lung fibrosis was analyzed by western blot, and (g) relative protein levels were quantified. ^∗^*P* < 0.05, ^∗∗^*P* < 0.01, ^∗∗∗^*P* < 0.001, and ^∗∗∗∗^*P* < 0.0001.

Bioinformatics analysis of IPF patients and BLM-induced pulmonary fibrosis mice were initially conducted to explore the differential gene expression in lung fibrosis tissues. Data obtained from tissue specimens of IPF patients and mice from GEO database (GSE77326, GSE2051) were investigated. It was found that the lung fibrosis tissues exhibited higher mRNA expressions of IL-19 compared with the normal lung tissues in both human and mice (Figure 1(a)). Following in vivo, we tested whether IL-19 was also upregulated in the well-established BLM-induced murine fibrosis models. C57BL/6 mouse lungs were acquired following the intratracheal injection of either saline or BLM (2.5 mg/kg) on day 21 [23]. We performed histological examination of lung tissue, including H&E staining, Masson's trichrome staining and IL-19 immunohistochemical staining (Figure 1(b)). The degree of lung fibrosis was quantified on the basis of modified ashcroft scale and lung hydroxyproline content (Figure 1(c)). The results demonstrated that BLM-induced thickening of the major tracheal wall and higher collagen contents in interstitial tissues, indicating the successful model construction. IL-19 immunohistochemical staining revealed the higher IL-19 expressions in lung fibrosis tissues (Figure 1(d)), as well as alpha smooth muscle Actin (*α*-SMA). Besides, we found BLM-induced mice had elevated levels of IL-19 in peripheral blood compared with control groups by ELISA (Figure 1(e)). The western blot analysis of lung tissues showed increased expressions of IL-19 in BLM-induced mice when compared with controls (Figures 1(f) and 1(g)).

### 3.2. IL-19 Is a Profibrotic Cytokine by Activating Fibroblast in Lung

**Figure 2:**
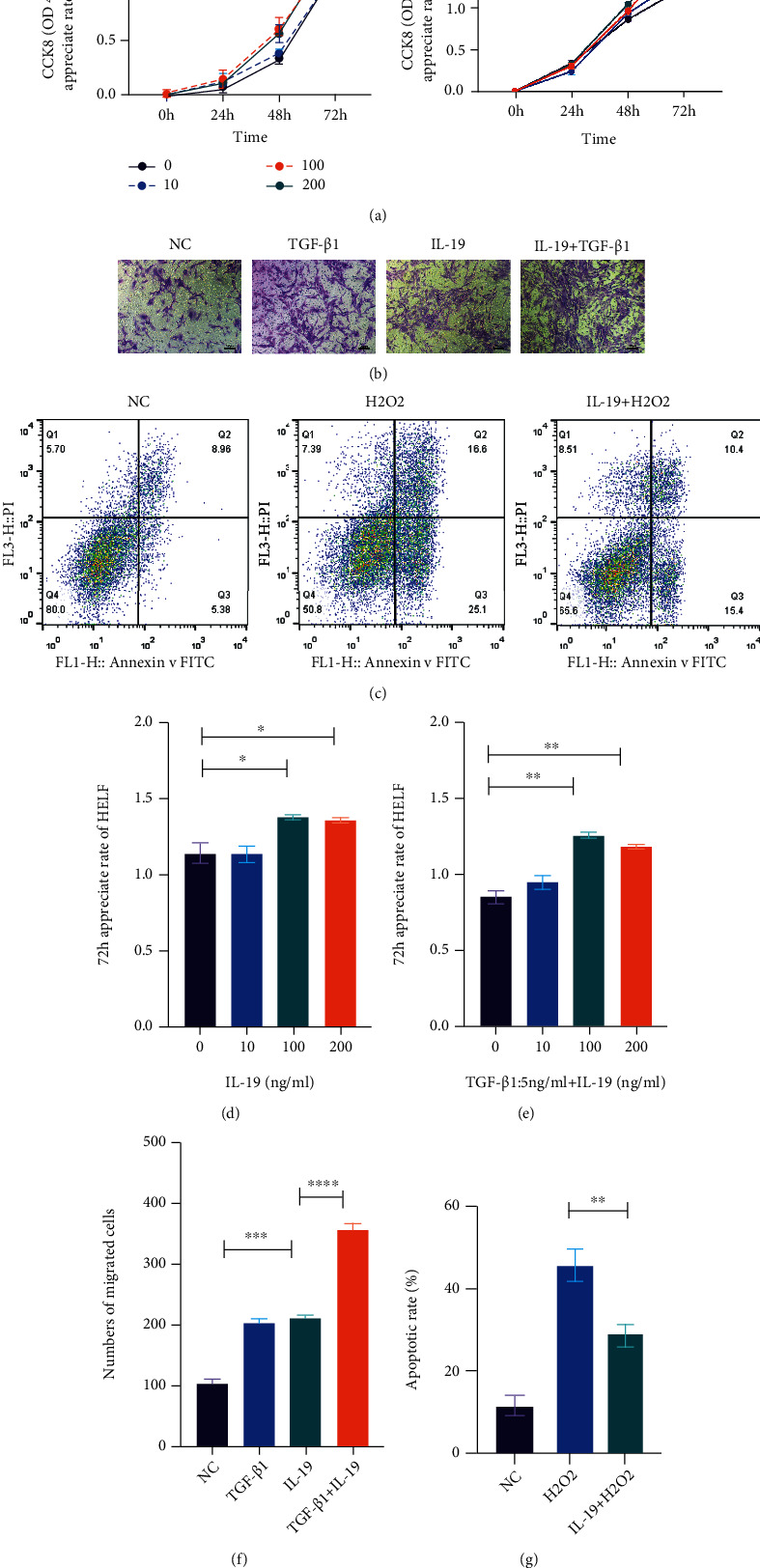
IL-19 promotes proliferation and invasion, while represses apoptosis of lung fibroblasts. (a) Lung fibroblasts appreciate rates were assessed by CCK-8 assay at 0 h, 24 h, 48 h, and 72 h with different IL-19 concentration of 0, 10, 100, and 200 ng/ml, and combination TGF-*β*1 (5 ng/ml) of different IL-19 concentration (0, 10, 100 and 200 ng/ml), and 72 h cell appreciate rates were analyzed (d and e). (b) Lung fibroblasts were, respectively, treated with TGF-*β*1 (5 ng/ml), IL-19 (100 ng/ml), IL-19 + TGF-*β*1 (5 *ng*/*ml* + 100 *ng*/*ml*), cell invasions were evaluated by transwell assay, and numbers of migrated cells were quantified (f). (c) The degrees of apoptosis were assessed by flow cytometry using propidium iodide and Annexin V staining, and apoptosis rates were calculated (g). ^∗^*P* < 0.05, ^∗∗^*P* < 0.01, ^∗∗∗^*P* < 0.001, and ^∗∗∗∗^*P* < 0.0001.

To clarify whether IL-19 is pro fibrotic or antifibrotic in the lung, we first explored the effect of IL-19 on the biological behavior of lung fibroblasts. Cell proliferation and migration abilities were analyzed in HELF and primary mouse lung fibroblasts. We evaluated the proliferation abilities of lung fibroblasts exposed to IL-19 by CCK8 assay, while TGF-*β*1 was used to be the positive control (Figure 2(a)). Cells proliferation rates were calculated at 0 h, 24 h, 48 h, and 72 h with different IL-19 concentrations of 0, 10, 100, and 200 ng/ml. We found cells growth were significantly promoted after exposure at the concentration of 100 ng/ml for 72 h (Figure 2(d)), while combination of TGF-*β*1 (5 ng/ml) and IL-19 (100 ng/ml) showing a higher appreciated rate, which provided the optimal concentration and time for further studies (Figure 2(e)). The transwell assay indicated that cells invasion abilities increased after IL-19 treatment and further enhanced along with TGF-*β*1 (Figures 2(b) and 2(f)). Furthermore, flow cytometry analysis of FITC/PI showed that IL-19 suppressed the apoptosis rate induced by H_2_O_2_ in lung fibroblasts (Figures 2(c) and 2(g)).

**Figure 3:**
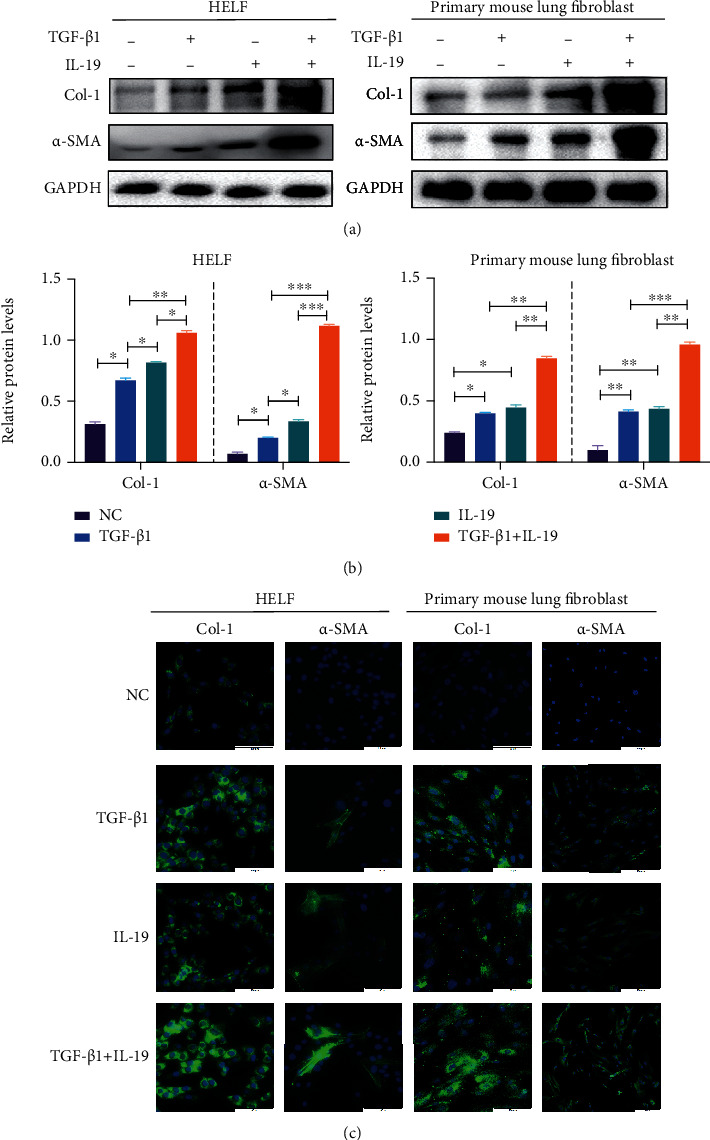
IL-19 promotes differentiation and collagen synthesis of lung fibroblasts. Proteomic analyses of Col-1 and *α*-SMA in HELF and primary mouse lung fibroblast treated by IL-19 (100 ng/ml) with or without TGF-*β*1 (5 ng/ml) by western blot (a), quantitative relative protein levels (b), and immunofluorescence staining (c). NC: negative control. ^∗^*P* < 0.05, ^∗∗^*P* < 0.01, ^∗∗∗^*P* < 0.001, and ^∗∗∗∗^*P* < 0.0001.

In addition, epithelial-mesenchymal transition (EMT) and fibrotic markers were analyzed in lung fibroblasts. We incubated lung fibroblasts with IL-19 (100 ng/ml, 72 hours) with or without TGF-*β*1 (5 ng/ml), then detecting protein expression of *α*-SMA and Col-1 by western blot. As seen in Figures 3(a) and 3(b), expression levels of *α*-SMA and Col-1 were elevated after IL-19 or TGF-*β*1 stimulation, and further elevated when combined IL-19 with TGF-*β*1. Immunofluorescence staining analysis also demonstrated the increase of *α*-SMA and Col-1 by IL-19 stimulation. We observed the highest intensity of fluorescence staining of *α*-SMA and Col-1 in IL-19 + TGF-*β*1 stimulation groups (Figure 3(c)).

### 3.3. IL-19 Promotes Lung Fibrosis through TGF-*β*/Smad Cascade

**Figure 4:**
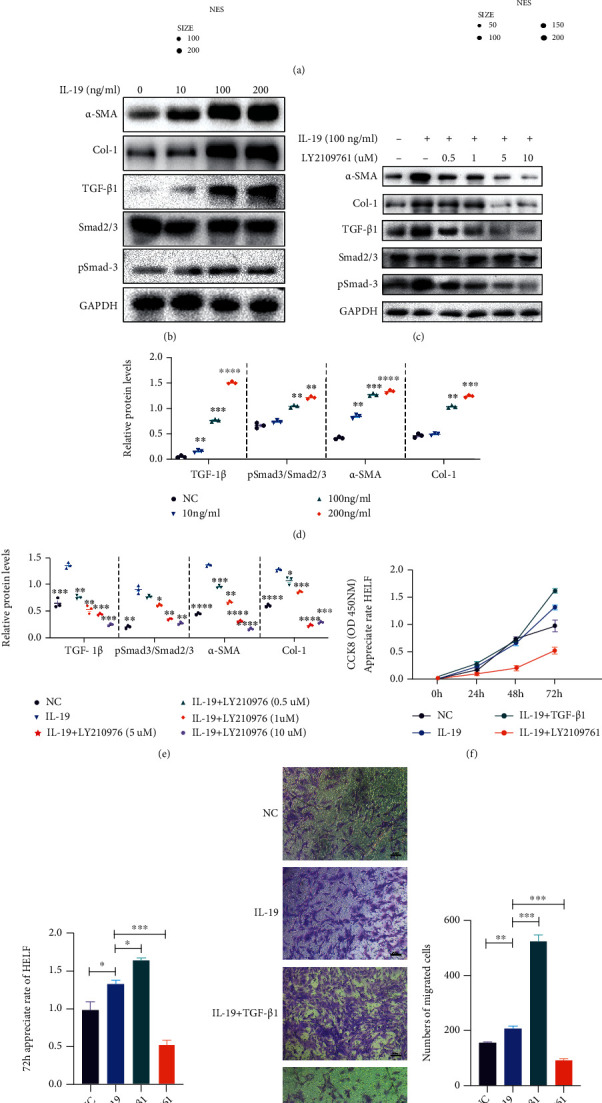
TGF-*β*1 blocker treatment attenuates expression and function of profibrotic markers in IL-19-stimulated lung fibroblasts. (a) The bioinformatics KEGG functional enrichment analysis of IL-19 in IPF patients and BLM-induced pulmonary fibrosis mouse lung tissues. (b) the western blots were used to analysis the expression of TGF-*β*1, Smad2/3, p-Smad3, Col-1, and *α*-SMA in lung fibroblasts treated with increased IL-19 concentration of 0, 10, 100, and 200 ng/ml, and (d) relative protein levels were quantified. Data were compared with NC group. (c) IL-19-stimulated lung fibroblasts treated with LY2109761 at concentration of 0.5–10 *μ*M for 72 h, and (e) relative protein levels were quantified. Data were compared with IL-19 group. (f) Lung fibroblasts were treated with IL-19 (100 ng/ml) or *IL* − 19 (100 *ng*/*ml*) + *TGF* − *β*1 (5 *ng*/*ml*) or IL-19 (100 *ng*/*ml*) + *LY*2109761 (10 *μM*), cell appreciate rates were assessed by CCK-8 assay at 0 h, 24 h, 48 h, and 72 h, and (g) 72 h cell appreciate rates were analyzed. (h) Cell invasions were analyzed by transwell assay and (i) numbers of migrated cells were quantified. ^∗^*P* < 0.05, ^∗∗^*P* < 0.01, ^∗∗∗^*P* < 0.001, and ^∗∗∗∗^*P* < 0.0001.

TGF-*β*/Smad signaling has a central role in the development of pulmonary fibrosis that drives activation of myofibroblasts (MFs), excessive production of ECM, and inhibition of ECM degradation [24]. TGF-*β*1 phosphorylates Smad2/3 and regulates target gene expression [25]. Above results showed the effect of IL-19 on biological behaviors of fibroblast could be enhanced along with TGF-*β*1, implying a potential interactive relationship between them. To identify significantly dysregulated biological pathways of IL-19 in IPF lung tissues, we performed bioinformatics analysis of GSEA under functional annotations of the KEGG database. KEGG functional enrichment analysis showed that TGF-*β*/Smad pathway enriched in human and mouse lung fibrosis and the expression of IL-19 in IPF was positively correlated with the profibrosis critical TGF-*β*/Smad signaling pathway (Figure 4(a)). Accordingly, to investigate the impact of IL-19 on TGF-*β*/Smad signaling pathway, we treated fibroblasts with increased concentration of IL-19 (0, 10, 100, and 200 ng/ml) and measured TGF-*β*1, Smad2/3 and phospho-Smad3 (pSmad3) expressions by western blot. The results showed the elevated expression levels of TGF-*β*1, pSmad3/Smad2/3 along with IL-19 concentration growth, demonstrating the activation of TGF-*β*/Smad cascade pathway induced by IL-19 (Figures 4(b) and 4(d)).

LY2109761 is a TGF-*β* type I/II receptor kinase inhibitor that suppressing the phosphorylation of Smad2 and Smad3. To confirm that IL-19 could active TGF-*β*/Smad signaling pathway, we examined the rescued impact of LY2109761 on IL-19-stimulated lung fibroblasts. We treated fibroblasts with concentrations of 0.5–10 *μ*M of LY2109761 for 72 h. The proteomic analyses of TGF-*β*1, Smad2/3, and pSmad3 showed that the treatment of LY2109761 effectively blocked TGF-*β*/Smad cascade (Figures 4(c) and 4(e)). We further determined the effect of LY2109761 on the biological behaviors of IL-19-stimulated lung fibroblasts. Cells were treated with IL-19 (100 ng/ml) with or without LY2109761 (10 *μ*M) for 72 h. Cell viabilities were determined by the CCK8 assay. Figures 4(f) and 4(g) showed that the proliferation promotion effect of IL-19 on fibroblasts could be significantly inhibited by LY2109761. The transwell analysis showed that LY2109761 significantly suppressed the promotion of migration induced by IL-19 on lung fibroblasts (Figure 4(h)). In addition, LY2109761 inhibited secretion of *α*-SMA and Col-1 proteins in a concentration-dependent manner in IL-19-stimulated lung fibroblasts (Figures 4(c) and 4(e)), indicating LY2109761 suppressed the differentiation and collagen synthesis induced by IL-19 in lung fibroblasts. Our results indicated that TGF-*β*/Smad signaling was activated in IL-19-stimulated lung fibroblasts and profibrotic effect of IL-19 on lung fibroblasts could be inhibited if we blocked TGF-*β*/Smad signaling pathway.

### 3.4. IL-19 Aggravates Lung Fibrosis In Vivo

**Figure 5:**
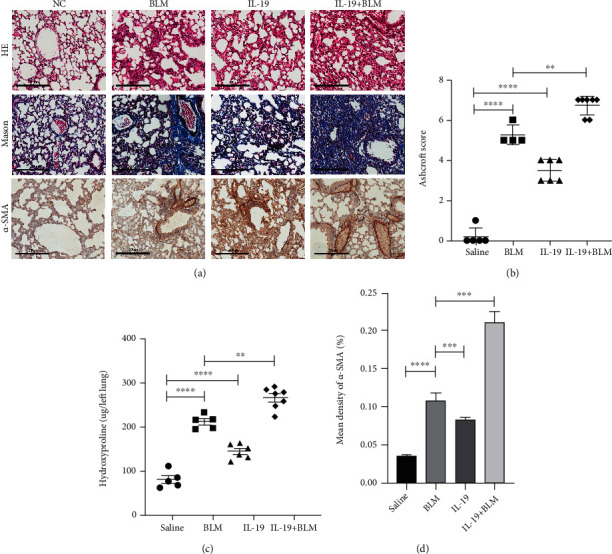
IL-19 aggravated lung fibrosis in wild-type and murine bleomycin fibrosis models. Schematic showing the induction of pulmonary fibrosis of lung collagen visualized by histopathological analysis after IL-19 and BLM challenge. After treatment with a single dose of BLM (2.5 mg/kg) or IL-19 (200 ng/kg) and combination of IL-19 and BLM, C57BL/6 mouse lungs were isolated and subjected for histopathology. (a) Representative pictures (20×) of H&E-stained, Masson's trichrome-stained sections and immunohistochemistry for *α*-SMA in lung sections. Effect of IL-19 on mice lung tissues were reflected by changes in the Ashcroft histology score (b) and lung hydroxyproline content (c). (d) Table represents the semiquantitative evaluation of protein expression (*α*-SMA) in specified treatment groups. In b–c, each symbol represents an individual mouse, a total of 5 to 7 mice per group. Scale bars, 200 *μ*m. ^∗^*P* < 0.05, ^∗∗^*P* < 0.01, ^∗∗∗^*P* < 0.001, and ^∗∗∗∗^*P* < 0.0001.

To determine the effect of IL-19 in progression of pulmonary fibrosis in vivo, we, respectively, administered a single dose of IL-19 (200 ng/kg) or BLM (2.5 mg/kg) or combination of these two treatments to wild-type C57BL/6 mice by intratracheal route. The lung tissues were collected on day 21. As shown in Figure 5(a), IL-19 treatment induced disruption of lung structure and collagen deposition in wild-type mice, while the extent of lung fibrosis (Figure 5(b)), lung hydroxyproline content (Figure 5(c)), and the expression of *α*-SMA (Figure 5(d)) were also higher than that in control groups. Furthermore, IL-19 treatment exacerbated BLM-induced abnormal lung changes with increased inflammatory cell infiltration and collagen deposition. Collectively, these studies demonstrated that IL-19 treatment could promote and aggravate the lung fibrosis progression induced by BLM in vivo.

## 4. Discussion

IL-19, as a new inflammatory factor in the regulation of the immune system, is related to the progression of many diseases, including autoimmune diseases, inflammation diseases and cancer [11, 26]. The debates on IL-19 being proinflammatory or anti-inflammatory factor have not yet been settled. The function of IL-19 in the inflammatory response depends on the cell type and disease model. The expression of IL-19 is reported increased in asthma and COPD patients, and shows the positive correlation with the progression of these diseases [16]. Bronchial epithelial cells from asthma and COPD patients express large amounts of IL-19, which is involved in allergic airway inflammation through the activation of group 2 innate lymphocytes (ILC2, 15] and induction of Th2-dominant immune response disorder [27]. Accordingly, we speculated IL-19 expression in respiratory diseases might be deleterious. However, none of studies explore the role of IL-19 in the etiopathogenesis of pulmonary fibrosis.

Usual interstitial pneumonia (UIP) is the histopathological marker of IPF, characterized by the conversion of fibroblasts to MFs, which are responsible for the production of ECM and excessive collagen deposition in the lung [28]. Although two drugs, pirfenidone and nidanib, have been approved for the therapies of IPF [29], the response to antifibrotic therapy exhibits significant heterogeneity, making it difficult for individual prognosis. The pathophysiology of IPF is the subject of ongoing research. As our understanding of cytokine-mediated regulation of fibrosis continues to increase, novel approaches are likely to promote development of fibrotic diseases treatment. The TGF-*β* superfamily of ligands [30] are well-known drivers of fibrosis, and IL-1–IL-17A–TGF*β* axis [31] and the type 2 cytokine (IL-4 and IL-13) response [32] have been considered as critical roles in the progression of fibrosis. Our study firstly demonstrates the profibrotic role for IL-19 through direct effects on lung fibroblasts through TGF-*β*/Smad pathway. We find IL-19 is upregulated in IPF patients' lung tissues and BLM-induced murine fibrosis models, and the stimulation of lung fibroblasts by IL-19 induces its proliferation and invasion, inhibits apoptosis and promotes its differentiation to myofibroblast phenotype, which can be revised by LY2109761, a TGF-*β*/Smad signaling pathway inhibitor. In vivo study, we determine that IL-19 aggravates lung fibrosis in wild-type mice and BLM-induced pulmonary fibrosis models.

IL-19, IL-20, IL-22, IL-24, and IL-26 are categorized in the IL-20 subfamily, the organ-specific effects of these cytokines are attributed to variation of their receptor heterodimers between tissues. IL-19, IL-20, and IL-24 target a specific two types of receptor complexes: IL-20RA/IL-20RB which predominantly localized in the lung, skin, testis, ovary, and placenta, leading to duplication of their target cell profiles and biological functions [6]. The three receptor subunits are expressed by resident effector cells of target organs, including keratinocytes [33], synovial fibroblasts [34], osteoclasts [35], vascular smooth muscle cells [8], and intestinal [36] and airway epithelial cells [15], and not on cells traditionally associated with the immune system [35]. MFs are the key effector cells of fibrosis diseases, characterized by *α*-SMA positive and lack of epithelial or endothelial markers [37]. IL-20 has been found to activate quiescent hepatic stellate cells (HSCs) and promote the proliferation, migration, and production of inflammatory cytokines, and deposition of ECM from activated HSCs. Anti-IL-20 receptor monoclonal antibody shows the protective effects on CCl4-induced liver injury mouse models. IL-20RA^−/−^ mice are resistant to CCl4-induced liver fibrosis [38]. Consistently, our data support the mechanism of fibroblasts activation by IL-19 that lung fibroblasts are converted to MFs, implying that targeting at IL-19 may be a potential treatment strategy for lung fibrosis.

Chronic inflammation is a common hallmark of fibrosis disease [39]. Immune cells and injured intrinsic cells of the affected organ release large amounts of inflammatory cytokines and growth factors to maintain chronic inflammation, promote MFs proliferation, and enhance ECM production [3]. The expression of IL-19 is initially detected in immune cells, including in monocytes, macrophages, and B cells [40]. Airway epithelial cells [16], synovial fibroblasts [34], keratinocytes [33], and vascular smooth muscle cells (VSMCs) [8] are subsequently confirmed to express IL-19. Our study demonstrates the activation of lung fibroblast induced by IL-19, however, whether immune response dysfunction involved in this progression is currently unclear. Moreover, chronic dysregulation of type II alveolar epithelial cells (AEC2s) is thought to be central of pathological mechanisms of fibrogenesis in IPF, most of epithelial cells in IPF lungs are abnormally activated and produce mediators to promote the amplification of myofibroblasts [41, 42]. Whether epithelial cells could produce IL-19 to participate the progression and pathogenesis of lung fibrosis is worthy of further study.

## 5. Conclusions

In conclusion, our study firstly highlights the deleterious role of IL-19 on development of pulmonary fibrosis by modulating fibroblasts through TGF-*β*/Smad pathway and reinforces its promise as a new therapeutic target for intervention in pulmonary fibrosis.

## Data Availability

The data used to support the findings of this study are included within the article.
